# Event‐Related Brain Potentials and Frequency‐Following Response to Syllables in Newborns and Adults

**DOI:** 10.1111/ejn.70418

**Published:** 2026-02-08

**Authors:** G. Danielou, E. Hervé, A. S. Dubarry, B. Desnous, C. François

**Affiliations:** ^1^ Aix‐Marseille University, CNRS, LPL Aix‐en‐Provence France; ^2^ Aix‐Marseille University, CNRS, CRPN Marseille France; ^3^ Pediatric Neurology Department APHM, La Timone Children Hospital Marseille France; ^4^ Aix‐Marseille University, INSERM, INS Marseille France

**Keywords:** FFR, MMN, newborns, pitch, place of articulation formant transition, syllables

## Abstract

Auditory event‐related brain potentials such as the mismatch negativity (MMN) and the frequency‐following response (FFR) allow exploring speech sound encoding along the auditory pathway. Here, we collected event‐related brain potential (ERP) and FFR neural responses to syllables in healthy full‐term newborns (*N* = 17, mean age = 3 days) and adults (*N* = 21, mean age = 22.7). Participants were passively exposed to alternating blocks of syllables presented at either fast or slow stimulation rates while we recorded electroencephalography (EEG). Specifically, blocks containing the synthetic /oa/ syllable alternated with “oddball” blocks containing three natural syllables differing in place of articulation (one standard /da/ and two deviants /ba/ and /ga/). At the FFR level, we found that 3‐day‐old newborns (i) exhibit an already functional encoding of vowel pitch, (ii) show an immature encoding of vowel formant structure, replicating previous observations. At the ERP level, the two deviants elicited clear MMN in the two groups, although with different topographies, suggesting an immature sensitivity to place of articulation in newborns. These results confirm the role of experience‐dependent developmental factors that may differentially shape FFR and ERPs of speech sound features. Furthermore, this study highlights the feasibility of assessing the hierarchy of neural speech sound encoding in a short experimental session.

## Graphical abstract

We collected ERP and FFR neural responses to syllables in 17 healthy full‐term newborns and 21 adults. Participants were passively exposed to alternating blocks of syllables presented at either fast or slow stimulation rates while we recorded electroencephalography. At the FFR level, newborns exhibit an already functional encoding of vowel pitch but an immature encoding of vowel formant structure. At the ERP level, two deviants elicited clear MMN in the two groups, although with different topographies, suggesting an immature sensitivity to place of articulation in newborns.

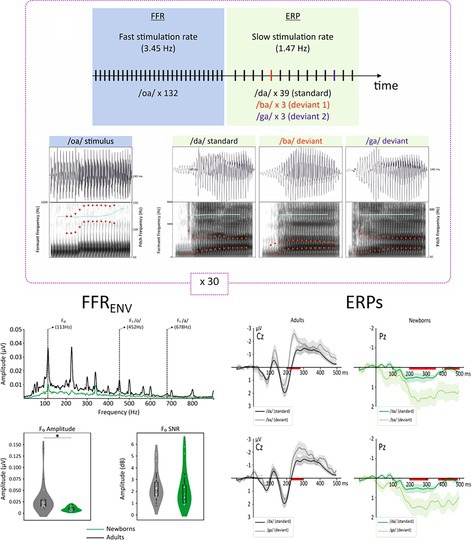

**Abbreviations**
ANOVA: analysis of variance
CIC: cascaded‐integrator‐comb
CV: consonant–vowel
EEG: electroencephalography
ERP: event‐related potential
F0: fundamental frequency
F1: first formant
F2: second formant
F3: third formant
FFR: frequency‐following response
FFRENV: enveloppe frequency‐following response
FFRTFS: fine‐temporal structure frequency‐following response
FSR: fast stimulation rate
GA: gestational age
IIR: infinite impulse response
IQR: interquartile range
MMN: mismatch negativity
MMR: mismatch response
RMS: root‐mean‐square
SNR: signal‐to‐noise ratio
SOA: stimulus‐onset asynchrony
SSR: slow stimulation rate
TW: time window

## 1 Introduction

Robust auditory processing abilities are crucial for acquiring complex linguistic functions during early development (Cabrera and Gervain 2020; Kujala et al. 2023). In the first year of life, the emergence of language‐specific developmental milestones is linked to sensitive periods of brain plasticity (Werker and Hensch 2015). The functional specialization of the speech network may thus depend on the interaction between genetic predispositions and environmental factors during these critical windows (Dehaene‐Lambertz et al. 2006; Gervain 2015). Importantly, the fundamental principles supporting the acquisition of speech sound categories may be linked to the brain's sensitivity to regularities of acoustic–phonetic cues in the auditory input, likely involving predictive coding and distributional learning mechanisms (Escera 2023; François and Schön 2014; Maye et al. 2002). However, even though auditory learning is present prenatally for some phonetic features (Moon et al. 2013; Partanen, Kujala, et al. 2013), it remains essential to determine whether newborns who have only a few hours of exposure to the environment encode subtle acoustic–phonetic cues in a similar way as adults do.

The perception of phonetic categories is an essential aspect of speech processing that relies on the sensitivity to subtle acoustic cues (Liberman et al. 1954). In French, stop consonants can be distinguished based on two main acoustic dimensions (Carranante et al. 2024). Voicing allows differentiating voiced (/b/, /d/, /g/) from unvoiced consonants (/p/, /t/, /k/) based on voice onset time, a fine‐grained temporal feature (Lisker and Abramson 1967). On the other hand, place of articulation allows differentiating labial (/b/, /p/), dental (/d/, /t/), and velar (/g/, /k/) stops based on the fast modulation of the second and third formants (F2 and F3) during the consonant–vowel transition period (Narayan 2019). Interestingly, young infants raised in English families already demonstrate behavioral sensitivity to voicing and place of articulation, suggesting that the encoding of these phonetic cues is functional early in development (Eimas 1974; Eimas et al. 1971; Moffitt 1971; Morse 1972; Walley et al. 1984). However, the degree of acoustic salience in the phonetic contrasts used to evaluate discrimination may explain why some contrasts are better perceived by adults than infants (Narayan 2019).

Thanks to recent advances in auditory neuroscience, research focusing on the brain correlates of speech sound acquisition has expanded considerably, particularly with event‐related brain potentials (ERPs) collected in newborns and infants (see Hervé et al. 2022 for a review). The mismatch negativity (MMN) is one of the most widely used ERP components, particularly well‐suited for investigating early speech perception (Cheour et al. 1998; Kuhl 2004; Rivera‐Gaxiola et al. 2000, 2005). In adults, the MMN is a frontocentral component that peaks around 200 ms after the onset of infrequent stimuli randomly inserted in a series of standard stimuli. The MMN may reflect the preattentive automatic detection of acoustic changes in a sequence of auditory stimuli (Kujala et al. 2023; Näätänen et al. 2007). The MMN is thus a valuable indicator of the brain's ability to detect auditory irregularities, including in infants and children whose behavioral responses are difficult to collect. The MMN is elicited by changes in pitch, duration, consonant voicing, or vowel identity in neonates and children (Cheour et al. 1998; Chobert et al. 2012, 2014; François et al. 2021; Partanen, Pakarinen, et al. 2013; Rivera‐Gaxiola et al. 2005). Importantly, young infants can exhibit a positive mismatch response (MMR) to phonetic changes (Cheng et al. 2015; Liu et al. 2023; Virtala et al. 2022) which is interpreted as an immature brain response (He et al. 2007, 2009; but see Govaart et al. 2023 for an alternative interpretation based on perceptual distance). Furthermore, newborns show MMRs to CV syllables differing in various phonetic features such as vowel identity, vowel pitch, vowel duration, and consonant voicing (Cheour‐Luhtanen et al. 1995; Chládková et al. 2021; François et al. 2021; Partanen, Pakarinen, et al. 2013). Only a few studies have explored the neural correlates underlying the discrimination of consonants based on place of articulation in neonates using the /ba/−/ga/ contrast (Dehaene‐Lambertz and Dehaene 1994; Mahmoudzadeh et al. 2016). However, to our knowledge, no studies have examined the neonatal brain sensitivity to both dental‐bilabial and dental‐uvular contrasts. Besides, ERPs reflect the synchronized activity of cortical neurons, leaving this measure “blind” to the activity of auditory neurons located earlier in the auditory pathway.

The frequency‐following response (FFR) is another neurophysiological marker of auditory processing that can be observed in newborns, infants, children, and adults (Anderson et al. 2015; Gorina‐Careta et al. 2021; Kraus et al. 2017; Ribas‐Prats et al. 2019; Skoe et al. 2015). The FFR was initially considered to be mainly generated by the inferior colliculus, although recent magnetoencephalographic studies have revealed that cortical generators also contribute to the FFR, particularly at lower frequencies (Bidelman and Powers 2018; Coffey et al. 2016, 2019, 2021; Gorina‐Careta et al. 2021; Skoe et al. 2015). Depending on the method used to compute the FFR, two variants of the response can be obtained, revealing the envelope FFR (FFR_ENV_) and the temporal fine‐structure FFR (FFR_TFS_). While the FFR_ENV_ provides information about the auditory system's ability to track the fundamental frequency (F0) associated with voice pitch, the FFR_TFS_ rather reflects the neural tracking of higher frequencies such as formants (Aiken and Picton 2008; Kraus et al. 2017; Krizman and Kraus 2019). Therefore, the FFR can provide an extensive snapshot of neural phase‐locking to the spectrotemporal features of complex auditory stimuli within the auditory pathway. Previous FFR studies using speech sounds have demonstrated that infants and newborns encode the vowel F0, suggesting that the subcortical encoding of pitch is already functional at birth even though it continues to mature up to at least 1 month of age (Ribas‐Prats et al. 2019, 2022; Arenillas‐Alcón et al. 2021; Jeng et al. 2011, 2016). On the other hand, the encoding of higher frequencies, such as the vowel first formant, seems to mature slowly during infancy and even until childhood (Ribas‐Prats et al. 2023; Anderson et al. 2015; Johnson et al. 2008). However, only a few studies have simultaneously collected ERPs and FFRs to speech sounds in adults (Bidelman 2015; Elmer et al. 2017; Calcus et al. 2022; Cheng and Zhao 2024), and, to our knowledge, none have yet explored both responses to speech sounds in newborns and adults.

Here, we explored the different stages of speech encoding by analyzing both the MMN and the FFR in a within‐participant design. While the MMN is a long‐latency ERP component reflecting the neural automatic discrimination of phonetic category changes, the FFR is considered an index of sustained neural phase‐locking to the spectrotemporal characteristics of the auditory signal. Therefore, we collected both types of neural responses to different speech sounds in newborns and adults during a single recording session containing blocks of fast and slow stimulation rates tailored to target FFR and ERP responses, respectively. Specifically, during blocks of fast stimulation rates (FSRs), we recorded the FFR to a two‐vowel/oa/speech stimulus previously shown to elicit robust subcortical responses in newborns and adults (Arenillas‐Alcón et al. 2021). We also collected ERPs to natural consonant–vowel (CV) syllables (/da/, /ba/, /ga/) differing in place of articulation during oddball blocks of slow stimulation rate (SSR). Considering previous FFR literature (Arenillas‐Alcón et al. 2021; Anderson et al. 2015; Jeng et al. 2011, 2016), we expected newborns to show similar encoding of F0 as adults but an immature encoding of the vowel formants. At the cortical level, considering previous findings in adults and newborns (Kraus et al. 1992; Dehaene‐Lambertz and Dehaene 1994), we expected significant MMNs over frontocentral electrodes to both types of deviants. However, we expected a positive MMR with a centroparietal topography in newborns probably reflecting an immature cortical processing.

## 2 Materials and Methods

### 2.1 Participants

A total of 38 participants were enrolled in the study with 17 healthy full‐term neonates (8 females; mean gestational age (GA) at birth in weeks = 39 weeks GA [38–40]; mean birth weight in grams = 3026 [1680–3820]; mean postnatal age at test in days = 2.6 [1–4]; Apgar‐5 > 7) and 21 healthy young adults (15 females; mean age in years = 24 [18–30]) with no hearing difficulties, no self‐reported history of neurological and psychiatric disease. Although relatively small, this sample size is comparable to those of prior studies analysing ERPs or FFRs in newborns or adults (François et al. 2021; François and Schön 2011; Jeng et al. 2011). All neonates had normal hearing from the universal screening test (automated auditory brain stem response) and normal examination made by a neonatologist at the delivery ward. All the newborns were recruited and tested at the Conception Hospital in Marseille (South of France), while adults were recruited through local advertisement at the university and tested at the Laboratoire Parole et Langage. The study has been approved by the SUD‐EST III ethical committee (2021‐A02615‐35), and informed consent was obtained from the caregivers or the participants at the beginning of the experimental session, in accordance with the Declaration of Helsinki.

### 2.2 Stimuli

**FIGURE 1:**
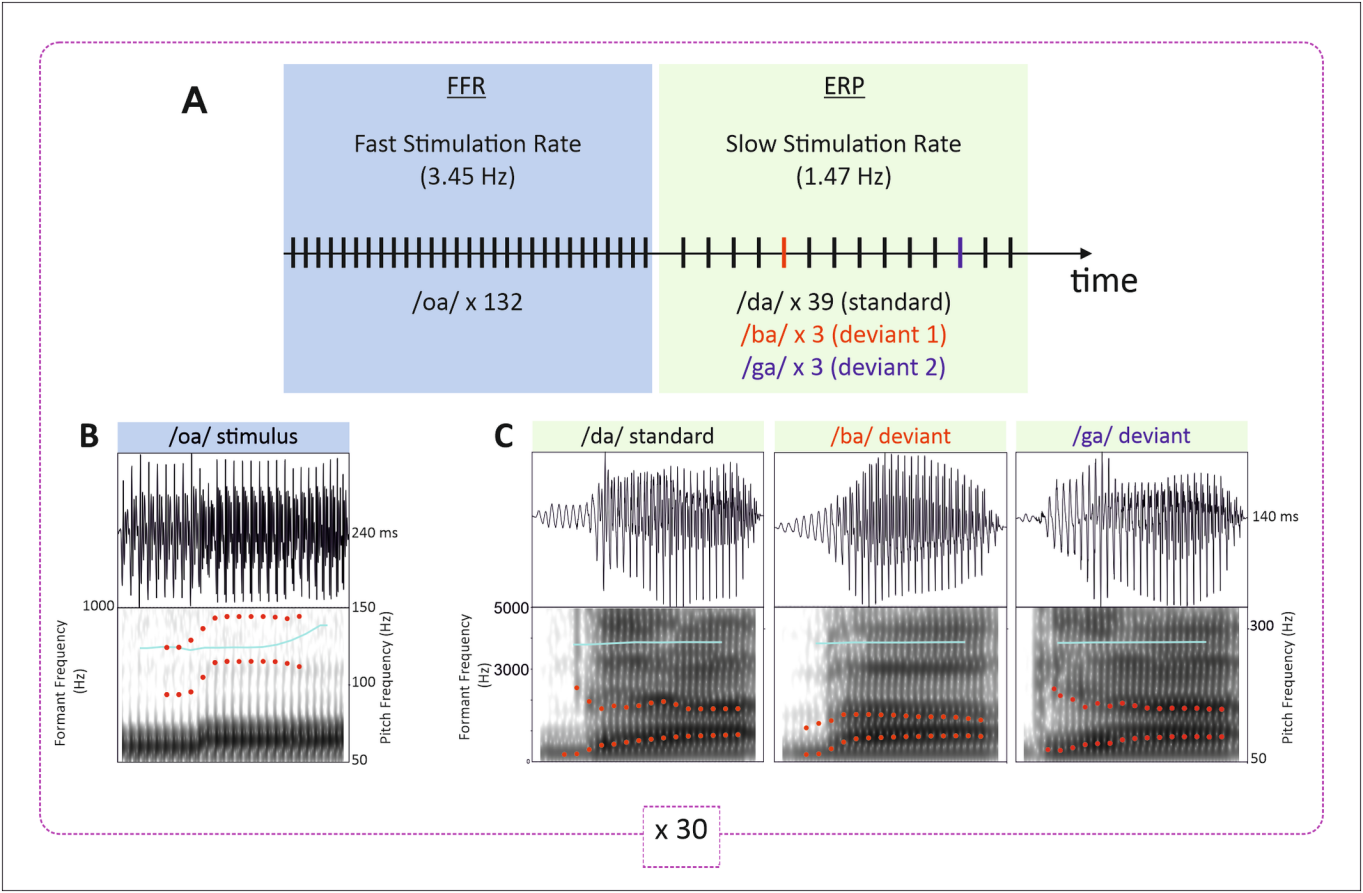
Experimental design used in the present study. (A) The stimulation sequence included blocks of fast stimulation rate (FSR) composed of 132 repetitions of the two‐vowel/oa/ stimulus that alternated with blocks of slow stimulation rate (SSR), creating oddball sequences with one/da/ standard and two deviants in place of articulation/ba/and/ga/. (B) Illustration of the/oa/stimulus (Arenillas‐Alcón et al. 2021) used in the FSR blocks with the waveform and spectrogram. (C) Illustration of the three CV syllables used in the SSR blocks. The red dots depict the first two formants (F1 and F2), and the blue trace depicts the fundamental frequency. Note the differences in F2 between the standard and the two deviants.

Stimuli were complex auditory sounds consisting of four syllables: one synthetic /oa/ and three natural /da/, /ba/, and /ga/ (see Figure 1). The /oa/ stimulus was a 240‐ms two‐vowel syllable with a pitch‐rise ending previously used to obtain robust FFR in newborns and adults (Arenillas‐Alcón et al. 2021). Briefly, the /o/ vowel period (F1 = 452 Hz; F2 = 791 Hz) lasted from 0 to 80 ms, the vowel period /a/ (F1 = 678 Hz; F2 = 1017 Hz) from 90 to 240 ms, and the /oa/ formant transition period from 80 to 90 ms. A fundamental frequency of 113 Hz remained stable from 0 to 160 ms and increased linearly to 154 Hz from 160 to 240 ms. The /da/, /ba/, and /ga/ sounds were natural consonant–vowel syllables recorded by a French female speech therapist and modified with Praat software. Each of the three syllables lasted 140 ms and had a fundamental frequency of 250 Hz. The values of the first formant (F1) were extracted from the steady‐state part of the vowel, while those of the second formant (F2) were obtained during the consonant–vowel transition (see Table 1).

**TABLE 1:** Acoustic characteristics of the stimuli used in the present study. For each syllable, the values of F1 and F2 formants were extracted at the onset and offset of the CV transitions with the software Praat.

	F1 dynamics (Hz) during CV transition (onset–offset)	F2 dynamics (Hz) during CV transition (onset–offset)
**Standard/da/**	304–528	1955–1755
**Deviant/ba/**	378–567	1381–1447
**Deviant/ga/**	369–588	2141–2116

### 2.3 Procedure

For newborns, the entire recording session took place in a quiet room of the maternity ward at Conception Hospital with at least one parent present. For adults, the recording session took place at the Laboratoire Parole et Langage. Infants were tested while sleeping in their cribs or their parents' arms. Adults sat in a comfortable chair while watching a subtitled silent video. Adult participants were asked to watch the movie without focusing on the sounds. The passive listening paradigm was adapted from Bidelman (2015) and lasted 35 min. This procedure allows for recording both FFR and ERP responses, which require a different number of trials to be robustly extracted. Stimuli were delivered in 30 alternating blocks of fast (3.45 Hz) and slow (1.47 Hz) stimulation rates (see Figure 1), with a stimulus‐onset asynchrony (SOA) of 290 and 680 ms, respectively. In each of the FSR sequences, the /oa/ syllable was presented 132 times in alternating polarities leading to a total of 3960 /oa/ trials. This procedure allowed adding the neural responses to the two polarities to isolate the FFR_ENV_ by minimizing stimulus artifact and cochlear microphonics (Aiken and Picton 2008; Chimento and Schreiner 1990). In the SSR sequences, a series of 45 CV syllables was presented with the standard /da/ in alternating polarities and two types of deviants, /ba/ and /ga/, without alternating polarity. SSR sequences were created according to multifeature oddball paradigms (François et al. 2021; Partanen, Pakarinen, et al. 2013) with 39 standards and six deviants in each sequence leading to a total of 90 trials for each deviant type and 1170 standard trials (three repetition of each of the two deviant types per block; 6.67% probability for each deviant). The /oa/ and the standard /da/ stimuli were presented in alternating polarities to allow the extraction of FFR to both types of stimuli but here; we focus on the FFR to /oa/ only. Stimuli were delivered to the right ear (Skoe and Kraus 2010) at a volume of 70 dB via a shielded ER‐2 tubular insert in‐ear monitor (Etymotics ER 2; Etymotic Research Inc., Elk Grove Village, IL, USA) via a custom MATLAB script. Volume was precalibrated with a Bruel and Kjaer artificial ear (model 4152) and a 2250‐light‐G4 sound level meter, and monitored during data recording using a Fireface UCX sound card (RME, Haimhausen, Germany). To achieve the high temporal accuracy required for FFR recordings, triggers were included in the second channel of the stereo audio track, thus containing the auditory stimulus in the first channel and the trigger in the second. A splitter was used to send the stimulus to the inserted earphone on one side and the trigger to the BIOSEMI receiver on the other, as previously done (Calcus et al. 2022). The inserted earphone and the stimulation cable were shielded to minimize electrical leakage between the stimulation equipment and the scalp electrodes.

### 2.4 EEG Data Acquisition and Preprocessing

The EEG signal was recorded at a sampling rate of 16.384 Hz using a high‐speed firmware Biosemi amplifier system (Biosemi ActiveTwo, Amsterdam University) from 16 active Ag‐Cl scalp electrodes mounted on an elastic cap at standard 10/20 positions (Fp1, Fp2, F3, F4, T7, C3, C4, T8, P3, P4, O1, O2, Fz, Cz, Pz, and Oz). In the Biosemi system, the ground and reference electrodes used in conventional EEG systems are replaced by the Common Mode Sense active electrode and the Driven Right Leg passive electrode that were located at C1 and C2 sites, respectively. The 16 active electrodes were recorded with an online DC‐3.3‐kHz low‐pass filter (−3‐dB fifth‐order CIC low‐pass filter). Additionally, three electrodes were placed on the right and left mastoids for offline rereferencing and at the Fpz location for capturing the FFR. These three electrodes were recorded with an online DC‐3.3‐kHz low‐pass filter. Furthermore, the signal collected by the two mastoïds was also saved in a high frequency version with a first‐order high‐pass filter with −3 dB at 100 Hz (i.e., 100–3.3‐kHz bandwidth).

ERP data were analyzed using custom Python scripts incorporating functions adapted from the MNE library (Gramfort 2013). FFR data were analyzed using custom scripts based on MATLAB version R2022b (TheMathWorks Inc., 2022) and adapted functions of the Brain stem Toolbox (Skoe and Kraus 2010).

For FFR preprocessing, continuous EEG data gathered during the FSR sequences were offline filtered between 80 and 1500 Hz (Second‐order IIR Butterworth bandpass filter) and segmented into epochs from −40 to 270 ms from stimulus onset. Following baseline correction, epochs exceeding ±30 μV were automatically rejected. Then, because we presented the/oa/ stimulus in both the original and inverted polarities, we were able to extract the FFR_ENV_ which was obtained by averaging the responses elicited by the two opposite/oa/stimulus polarities [(Rarefaction + Condensation)/2], and the FFR_TFS_, which was obtained by subtracting the responses elicited by the two polarities [(Rarefaction − Condensation)/2]. After artifact rejection, the mean number of epochs was 3954 (SD = 10, [3922–3960]) for newborns and 3920 (SD = 43, [3796–3959]) for adults. The mean number of FFR epochs was significantly higher in newborns than in adults (U_(36)_ = 46, *p* < 0.001).

For ERP preprocessing, EEG data were re‐referenced offline to the algebraic average of the mastoids. Then, the continuous data were inspected visually to identify bad channels. Bad channels were interpolated when no more than two channels were detected. The whole dataset was excluded from further analyses when more than two bad channels were identified. Then, continuous EEG data recorded during the SSR sequences were downsampled to 256 Hz, offline filtered between 1 and 30 Hz (second‐order IIR Butterworth bandpass filter) and segmented into epochs from −100 to 500 ms from stimulus onset separately for each condition. Following baseline correction, epochs were automatically rejected when they exceeded ±150 μV in newborns and ±70 μV in adults. The first three standard trials of each SSR sequence were systematically removed from the analyses to neutralize the effects of novelty and provide a sufficient number of trials to create a short‐term memory trace (Näätänen et al. 2007). Finally, an additional rejection of epochs was performed manually by visually inspecting the remaining trials. Recordings with fewer than 150 standard (/da/) and 25 deviant trials (/ba/or/ga/) were excluded from the analyses as done in previous infants' EEG studies (Dehaene‐Lambertz and Dehaene 1994; Kabdebon et al. 2015; Urbanec et al. 2024). Individual averages were calculated for the standard (/da/) and for each deviant (/ba/and /ga/), and group averages were obtained. The difference wave was obtained for each deviant by subtracting the mean response to the standard from the mean response to the deviant to obtain the MMN. After artifact rejection, the mean number of standard (/da/) trials kept in the analyses was 829 (SD = 138, [522–1045]) for newborns and 806 (SD = 143, [583–1020]) for adults (t_(36)_ = 0.50, *p* = 0.622). For the /ba/ deviant, the mean number of trials was 72 (SD = 12, [46–84]) for newborns and 68 (SD = 12, [44–87]) for adults (U_(36)_ = 217, *p* = 0.264). For the /ga/ deviant, the mean number of trials was 70 (SD = 12, [47–87]) for newborns and 68 (SD = 12, [42–85]) for adults (t_(36)_ = 0.64, *p* = 0.529).

#### 2.4.1 FFRs

##### 2.4.1.1 Time Domain

###### 2.4.1.1.1 Root‐Mean Square (RMS)

We estimated the magnitude of neural activation over time by computing the RMS (Liu et al. 2015). The RMS was calculated by squaring each time point within a time period of the neural response, obtaining the mean of the squared values, and finally computing the square root. The RMS was obtained separately during the prestimulus (−40–0 ms) and the entire poststimulus period (0–240 ms).

###### 2.4.1.1.2 Neural Lag

Neural offset was used to estimate FFR latency, reflecting the transmission delay induced by the neural conduction velocity of the auditory system (Arenillas‐Alcón et al. 2021). Neural lag values were obtained by calculating the cross‐correlation between the stimulus and the FFR after bandpass filtering at 80–300 Hz, which isolates the pitch component (F0) to which FFRs preferentially phase‐lock and improves the robustness of F0 tracking measurements (Ananthakrishnan et al. 2021; Krizman and Kraus 2019; Guo et al. 2020). First, an *r*‐value was calculated to determine the degree of correlation between the two signals as a function of time lag between 3 and 13 ms (Arenillas‐Alcón et al. 2021; Ribas‐Prats et al. 2019, 2022, 2023) with the highest absolute *r*‐value. To perform the cross‐correlation, the stimulus was downsampled to 16,384 Hz to match the neural response, as recommended (Skoe and Kraus 2010). Since the values obtained did not follow a normal distribution, we used the Mann–Whitney *U* test to assess the significance of differences between groups.

###### 2.4.1.1.3 Pitch Error and Pitch Strength

The accuracy of F0 tracking was assessed by calculating a cross‐correlation between the F0 contour of the stimulus and the F0 contour of the brain response for each stimulus section. Pitch error and pitch strength were extracted from the normalized autocorrelation of the neural response and that of the stimulus, in 50‐ms sliding bins. This analysis was performed in the time windows corresponding to the stable (0–160 ms) and the rising part of the stimulus (160–240 ms). For the brain response, the neural offset (estimated with the neural lag) was added at the beginning of the time window. The pitch error corresponds to the mean absolute Euclidean distance between these two F0 contours, with lower values indicating more accurate pitch tracking. While pitch error measured the accuracy of F0 encoding, pitch strength was used as a measure of the extent of neuronal phase‐locking (periodicity) to the stimulus F0. Because data were not normally distributed, we used a Mann–Whitney *U* test to assess potential between‐group differences using group (newborns vs. adults) as a grouping variable and stimulus section (/a/ stable vs. /a/ rising) as contrast variable. To examine within‐participant differences (i.e., whether stimulus–response correlations varied according to stimulus section), a Wilcoxon paired‐samples test was conducted, comparing the correlation values obtained for each stimulus section (/a/ stable vs. /a/ rising). Finally, we conducted a second Mann–Whitney *U* test with group as the between‐participant factor and the difference between the two stimulus sections (/a/stable—/a/rising) as the within‐participant factor, to test for a possible interaction.

##### 2.4.1.2 Frequency Domain

FFR features in the frequency domain were obtained to assess participants' encoding of the F0 and the formant structure of the speech sound/oa/. More specifically, analyses focused on the encoding of the F0 (113‐Hz constant section and 113–154 Hz rising pitch ending) obtained from the analysis of the FFR_ENV_ and the first formant/o/ (0–80 ms; 452 Hz); /a/section (90–240 ms; 678 Hz) obtained from the analysis of the FFR_TFS_.

###### 2.4.1.2.1 Spectral Amplitude at Peak Frequency (F0 and F1)

Fast Fourier transforms (FFT) of the neuronal response for /o/ (10–80 ms) and /a/ (90–160 ms) were obtained. Next, spectral amplitudes at the F0 peak (113 Hz) were extracted from the FFR_ENV_ during the constant pitch section (10–160 ms). The F1 peak /o/ (452 Hz) and F1 peak /a/ (678 Hz) were extracted from the FFR_TFS_. Spectral amplitude was taken as an indicator of the extent of neural phase‐locking at the three frequencies of interest. As F0 amplitudes were normally distributed, a two‐sample *t*‐test was used to assess differences between groups. Spectral amplitudes at the peaks corresponding to the F1 frequencies of the vowels /o/ (F1 at 452 Hz) and /a/ (F1 at 678 Hz) were extracted separately from the neural responses to the associated temporal segments: 10–80 ms for the /o/ section and 90–160 ms for the /a/ section. Since the data were normally distributed, we conducted an ANOVA with group (adults vs. newborns) as a between‐participant factor and stimulus section (/o/ vs. /a/) as a within‐participant factor.

###### 2.4.1.2.2 Signal‐to‐Noise Ratio (SNR) (F0 and F1)

The SNRs around the F0 peak (SNR_F0_) and the F1 peak (SNR_F1_) were used to estimate the accuracy of encoding the spectral content of the response. The SNR at the fundamental frequency peak (F_0_) was used as an estimate of the relative spectral magnitude of the response. This measure takes into account not only the amplitude of the signal at the frequency peak of interest (113 Hz) but also that of surrounding frequencies. To do this, we calculated the ratio between the mean amplitude in a ±5‐Hz window centered on F_0_ (i.e., from 108 to 118 Hz) divided by the mean amplitude in two lateral windows of 28 Hz each at ±19 Hz from F0 (i.e., from 80 to 108 Hz and from 118 to 146 Hz; Arenillas‐Alcón et al. 2021; Ribas‐Prats et al. 2019, 2022). A Mann–Whitney *U* test was used to assess group differences because the values obtained were not normally distributed.

The same procedure was applied to F1, using the same frequency windows for signal and noise to obtain the (SNR_F1_) for section /o/ (452 Hz) and for section /a/ (678 Hz). An ANOVA was performed, including two factors: group (adults vs. newborns) and stimulus section (/o/ vs. /a/).

#### 2.4.2 ERPs

As a first step, we used nonparametric cluster‐based permutation tests (Maris and Oostenveld 2007) separately for each deviant type (/ba/and/ga/) and group (newborns and adults) using the permutation_cluster_1samp_test function (*p* < 0.05, *α* = 0.05, 1000 permutations, two‐tailed) from the MNE Python library to identify temporal clusters and electrodes with significant differences between standard and deviants (Werwach et al. 2022). This analysis was conducted from 100 to 500 ms after stimulus onset on nine electrodes (F3, Fz, F4, C3, Cz, C4, P3, Pz, and P4).

In a second step, we evaluated whether the two MMNs differed within each group. Specifically, we compared the MMNs between conditions (/ba/ vs. /ga/) separately for each group using the permutation_cluster_1samp_test function (*p* < 0.05, *α* = 0.05, 1000 permutations, two‐tailed). This analysis was conducted from 100 to 500 ms after stimulus onset on nine electrodes (F3, Fz, F4, C3, Cz, C4, P3, Pz, and P4).

Finally, we compared the MMNs between groups using the permutation_cluster_test function (*p* < 0.05, *α* = 0.05, 1000 permutations, two‐tailed) separately for each deviant. This analysis was conducted from 100 to 500 ms after stimulus onset on nine electrodes (F3, Fz, F4, C3, Cz, C4, P3, Pz, and P4).

## 3 Results

### 3.1 FFR

**FIGURE 2:**
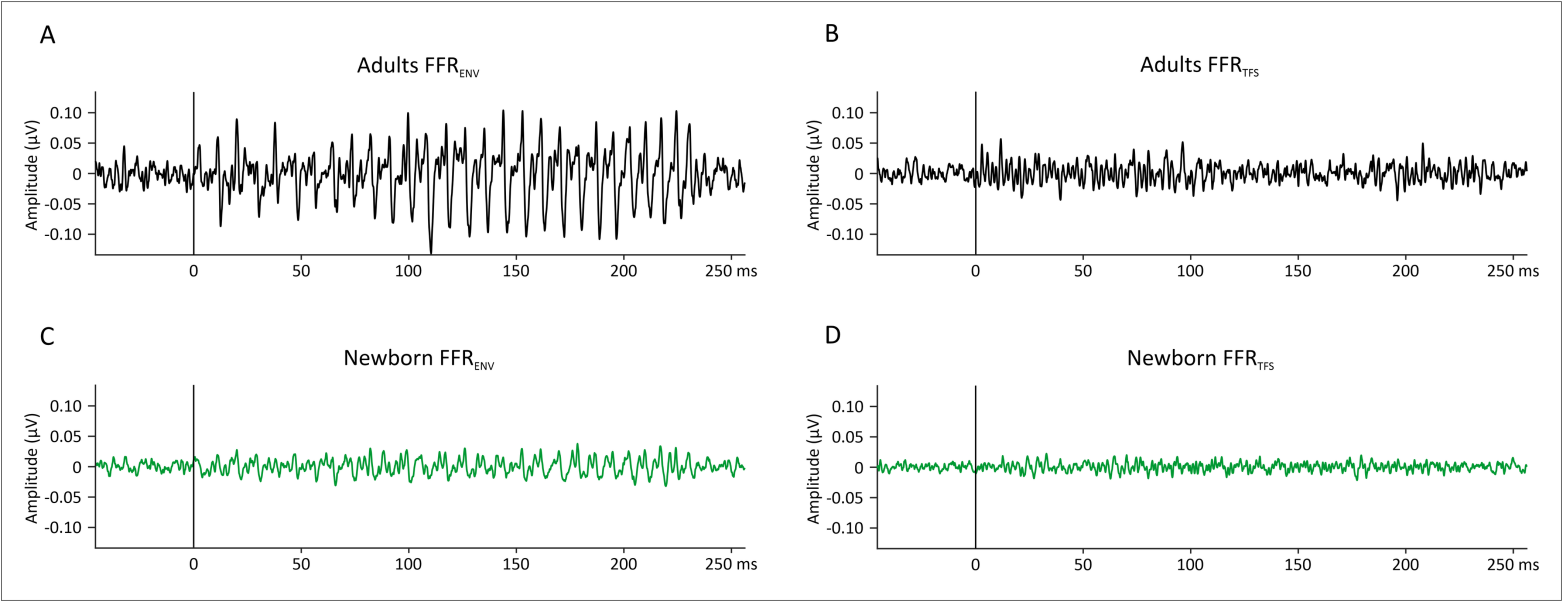
Grand‐average FFR waveforms in adults (top) and newborns (bottom) in response to the two‐vowel/oa/stimulus. The FFR_ENV_ was obtained by averaging the response to the two stimulus polarities while the FFR_TFS_ was obtained by subtracting the response to the two stimulus polarities. Note the larger amplitude of the response in adults compared to newborns.

As it can be seen on Figure 2, clear FFR responses were obtained in the two groups. Specifically, Figure 2 shows the grand‐average waveforms of the temporal envelope FFR obtained from averaged polarities (FFR_ENV_, Figure 2A,B) and temporal fine‐structure FFR obtained from subtracted polarities (FFR_TFS_, Figure 2C,D) in response to the two‐vowel/oa/separately for newborns and adults. As previously described (Arenillas‐Alcón et al. 2021), robust FFR_ENV_ and FFR_TFS_ waveforms were observed in both groups although with smaller amplitudes in newborns than in adults. This observation was confirmed when comparing the RMS between pre‐ and poststimulus periods. In both groups, the RMS_post‐stimulus_ was significantly higher than the RMS_pre‐stimulus_ (adults: W_(20)_ = 0, *p* < 0.001; newborns: W_(16)_ = 35, *p* = 0.050) suggesting that the stimulation induced a significant increase in neural response independently of age. Concerning the between group comparisons, the RMS_pre‐stimulus_ was significantly higher in adults (Mdn_adults_ = 0.047, IQR = 0.017) than in newborns (Mdn_newborns_ = 0.019, IQR = 0.005; U_(36)_ = 324, *p* < 0.001). Similarly, the RMS_post‐stimulus_ was significantly higher in adults (Mdn_adults_ = 0.073, IQR = 0.024) than in newborns (Mdn_newborns_ = 0.023, IQR = 0.012; U_(36)_ = 350, *p* < 0.001).

#### 3.1.1 Neural Lag

We evaluated differences in neural transmission velocity by comparing neural lag values between the two groups. As expected, newborns exhibited a longer neural transmission delay than adults [Mdn_newborns_ = 11.29 ms (IQR = 3.05), Mdn_adults_ = 9.46. ms (IQR = 2.01); U_(36)_ = 101.5, *p* < 0.05], reflecting the immaturity of the auditory pathway in newborns.

#### 3.1.2 Evaluation of F0 Encoding From the FFR_ENV_

##### 3.1.2.1 Spectral Amplitude at F0 Peak

**FIGURE 3:**
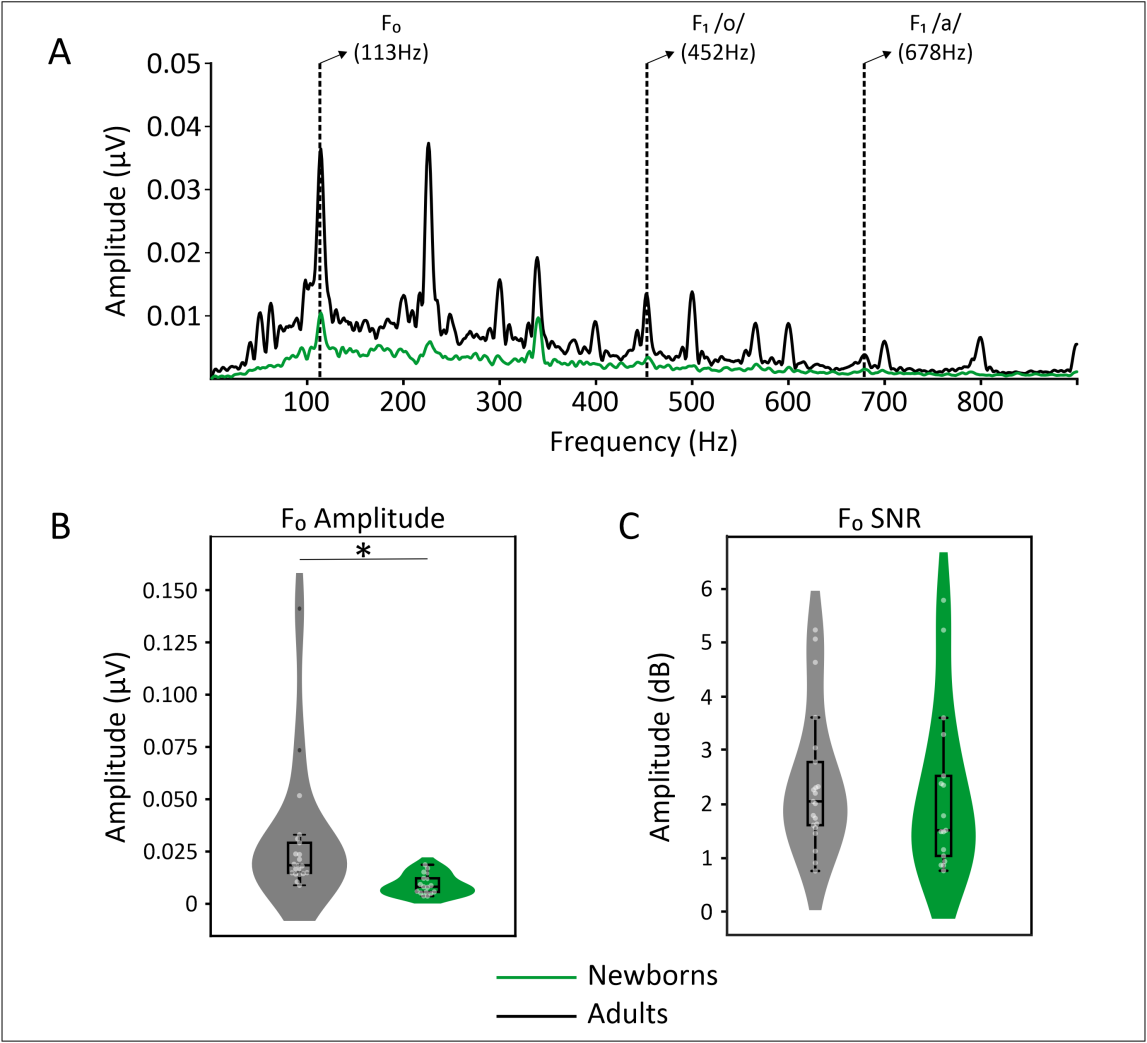
Grand‐average FFR_ENV_ amplitude spectra (A) in adults (black) and newborns (green) in response to the stable pitch section of the two‐vowel/oa/stimulus. Violin plots of the F0 amplitude (B) and F_0_SNR (C) in the two groups with individual values in gray. Note the significant F0 amplitude difference between adults and newborns.

To evaluate the encoding of the F0, we computed the amplitude spectra of the FFR_ENV_ for each group during the stable F0 section of the stimulus (i.e., 10–160 ms). As can be seen on Figure 3, clear peaks at the expected F0 target frequency (113 Hz) are observed for both groups, indicating efficient neural encoding of the F0. We evaluated between group differences by extracting the spectral amplitude at the F0 peak for each participant. Figure 3B depicts the distribution of these individual F0 spectral amplitude values in each group. Newborns showed significantly lower amplitudes at the F0 peak than adults [U_(36)_ = 320, *p* < 0.001].

##### 3.1.2.2 SNR at F0 Peak

The SNR at F0 peak was obtained to provide an estimate of the relative spectral amplitude of the response. As can be seen on Figure 3C showing the distribution of F0_SNR_ values obtained for each group, no significant between‐group difference was observed.

##### 3.1.2.3 Pitch Error

We examined the accuracy of F0 neural encoding for each stimulus section by computing the pitch error separately for each section and group. In the stable section, the Mann–Whitney *U* test evaluating differences between newborns and adults revealed no significant differences between groups (U_(36)_ = 148, *p* = 0.378). This was not the case for the rising section where a significant difference was observed with higher pitch error in newborns than in adults (U_(36)_ = 105, *p* = 0.032). Differences between sections were explored in each group using a Wilcoxon test. Results revealed a significant difference between the two sections (stable vs. rising/a/) in newborns (W_(16)_ = 19, *p* = 0.005, stable mean = 5.865; rising mean = 11.182) and adults (W_(20)_ = 38, *p* = 0.007, stable mean = 3.942; rising mean = 6.552). The second Mann–Whitney *U* test revealed no difference between groups and stimulus sections (U_(36)_ = 222, *p* = 0.207) suggesting that the accuracy of pitch tracking varies according to stimulus section, independently of group. This may reflect differences in the difficulty or stability of the pitch contour in these time windows.

##### 3.1.2.4 Pitch Strength

We examined the magnitude of neural phase‐locking by computing the pitch strength separately for each group and stimulus section. In the stable stimulus section, the Mann–Whitney *U* test evaluating differences between newborns and adults revealed no significant differences between groups (U_(36)_ = 236, *p* = 0.095). This was not the case for the rising section where a significant difference was observed with higher pitch strength in adults than in newborns (U_(36)_ = 262, *p* = 0.014). The Wilcoxon tests used to explore differences between sections in each group revealed no significant difference, newborns (W_(16)_ = 55, *p* = 0.329, stable mean = 0.386; rising mean = 0.433) and adults (W_(20)_ = 97, *p* = 0.539, stable mean = 0.530; rising mean = 0.564). The second Mann–Whitney *U* test revealed no difference between groups and stimulus sections (U_(36)_ = 187, *p =* 0.816).

#### 3.1.3 Evaluation of Formant Structure Encoding From FFR_TFS_

**FIGURE 4:**
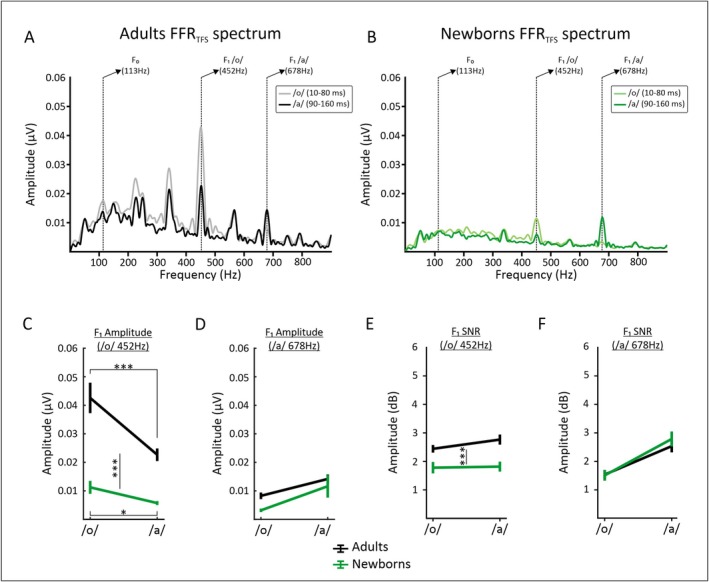
Grand‐average FFR_TFS_ amplitude spectra extracted from the/o/and/a/section in adults (A) and newborns (B). The target frequencies are indicated by vertical dashed lines. The means and standard errors for the F1 amplitude (C,D) and SNR (E,F) are shown for each stimulus section and group. * indicates significant differences with a *p*‐value < 0.05. *** indicates significant differences with a *p*‐value < 0.001.

We evaluated neural encoding of the first formant based on the extraction of the FFR_TFS_. As can be seen on Figure 4A,B, the grand‐average frequency spectra for the /o/ section and the constant pitch/a/ section show clear peaks at the expected target frequencies. The spectral amplitudes and SNRs of the FFR_TFS_ are shown in the two groups for the /o/ (10–80 ms) and the /a/ section (90–160 ms; see Figure 4C–F).

##### 3.1.3.1 Spectral Amplitude at/o/ Vowel F1 (452Hz)

The spectral amplitudes at the /o/vowel F1 are shown in Figure 4C. A significant main effect of group revealed larger spectral amplitude at 452 Hz in adults than in newborns [*F*_(1,36)_ = 33.70, *p* < 0.001, *η* ^2^_*p*_ = 0.48]. As expected, the main effect of stimulus section was also significant with larger spectral amplitude at 452 Hz in the /o/ than in the /a/ section [*F*_(1,36)_ = 32.20, *p* < 0.001, *η* ^2^_*p*_ = 0.47]. Most importantly, the group by stimulus section interaction was significant as well [*F*_(1,36)_ = 9.10, *p* = 0.005, *η* ^2^_*p*_ = 0.20].

Post hoc tests indicated that adults exhibited higher amplitude at 452 Hz for the /o/ than for the /a/ section (*t*_(20)_ = 5.03*, p* < 0.001, *d* = 1.10). Although significant, newborns showed a smaller effect than adults (*t*_(16)_ = 2.73, *p* = 0.03, *d* = 0.66).

##### 3.1.3.2 Spectral Amplitude at/a/ Vowel F1 (678Hz)

The spectral amplitudes at the /a/ vowel F1 are shown in Figure 4D. The main effect of stimulus section was significant and revealed higher spectral amplitude at 678 Hz in the /a/ than in the /o/ section [*F*_(1,36)_ = 14.14, *p* < 0.001, *η*_*p*_^2^ = 0.28]. The main effect of group was not significant nor was the group by stimulus section interaction (*p =* 0.45).

##### 3.1.3.3 SNR at/o/ Vowel F1 (452 Hz)

We also estimated the SNR of the/o/vowel F1 depicted in Figure 4E. The main effect of stimulus section was not significant. However, the main effect of group was significant [*F*_(1,36)_ = 13.42, *p* = 0.001, *η* ^2^_*p*_ = 0.27] while the group by stimulus section interaction was not (*p =* 0.177).

##### 3.1.3.4 SNR at/a/ Vowel F1 (678 Hz)

SNR values at the /a/ vowel F1 are visible on Figure 4F. The main effect of stimulus section was significant with larger SNR values in the /a/ than in the /o/ section [*F*_(1,36)_ = 45.7, *p* < 0.001, *η* ^2^_*p*_ = 0.56], suggesting a better encoding of the target frequency in the /a/ compared to the /o/ section. Neither the main effect of group nor the group by stimulus section interaction was significant.

### 3.2 ERPs

**FIGURE 5:**
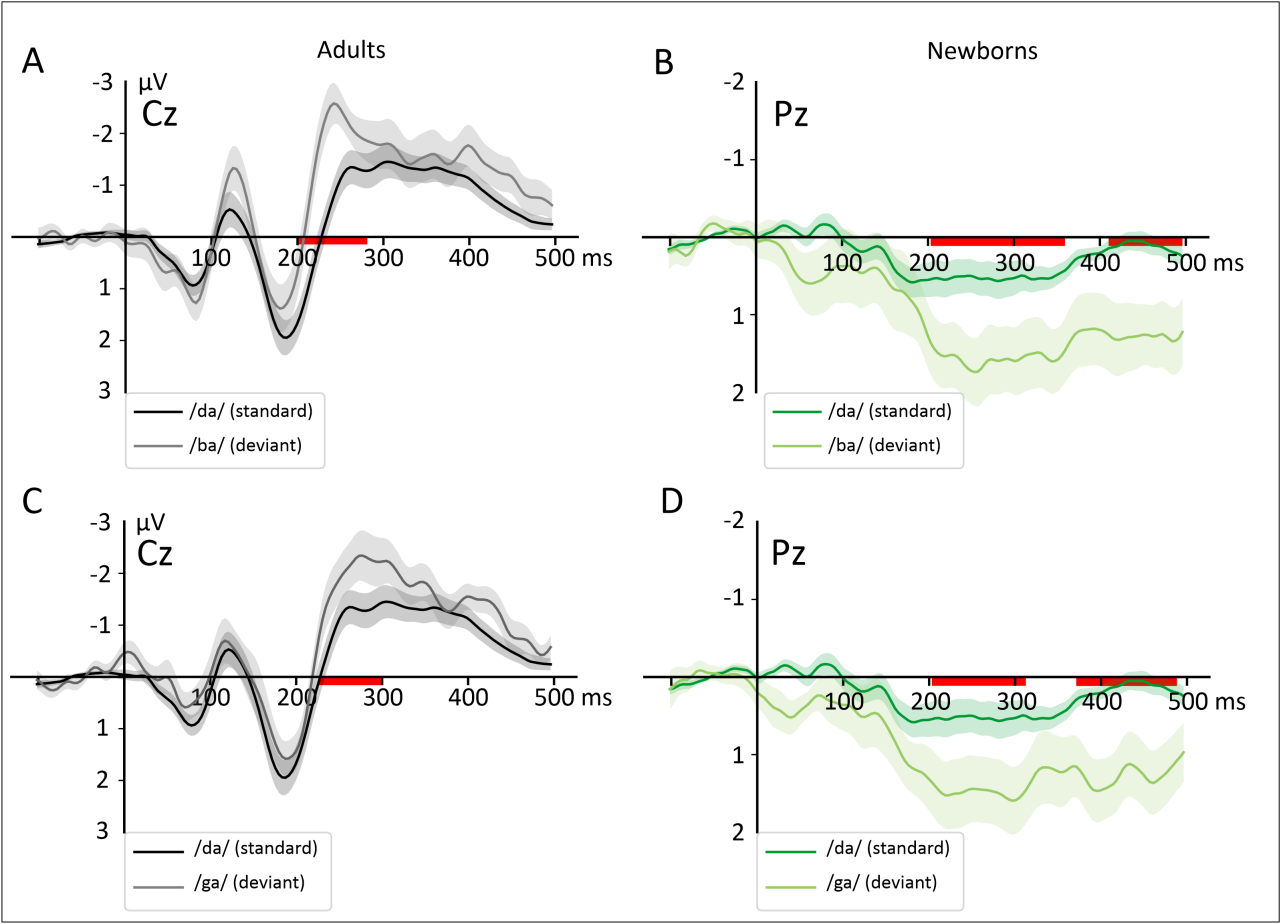
Grand‐average ERPs to standard and deviants in adults (left) and newborns (right) separately for each deviant and condition at Cz and Pz electrodes. ERPs to standards are shown in dark colors (dark black for adults and dark green for newborns). ERPs to deviants are shown in light colors (light gray for adults and light green for newborns) for the/ba/ (top) and/ga/deviant (bottom), respectively. The shaded areas around the curves depict the standard errors. The red horizontal bars show the temporal clusters with significant differences between standard and deviants at *p* < 0.05 corrected for multiple comparisons.

**FIGURE 6:**
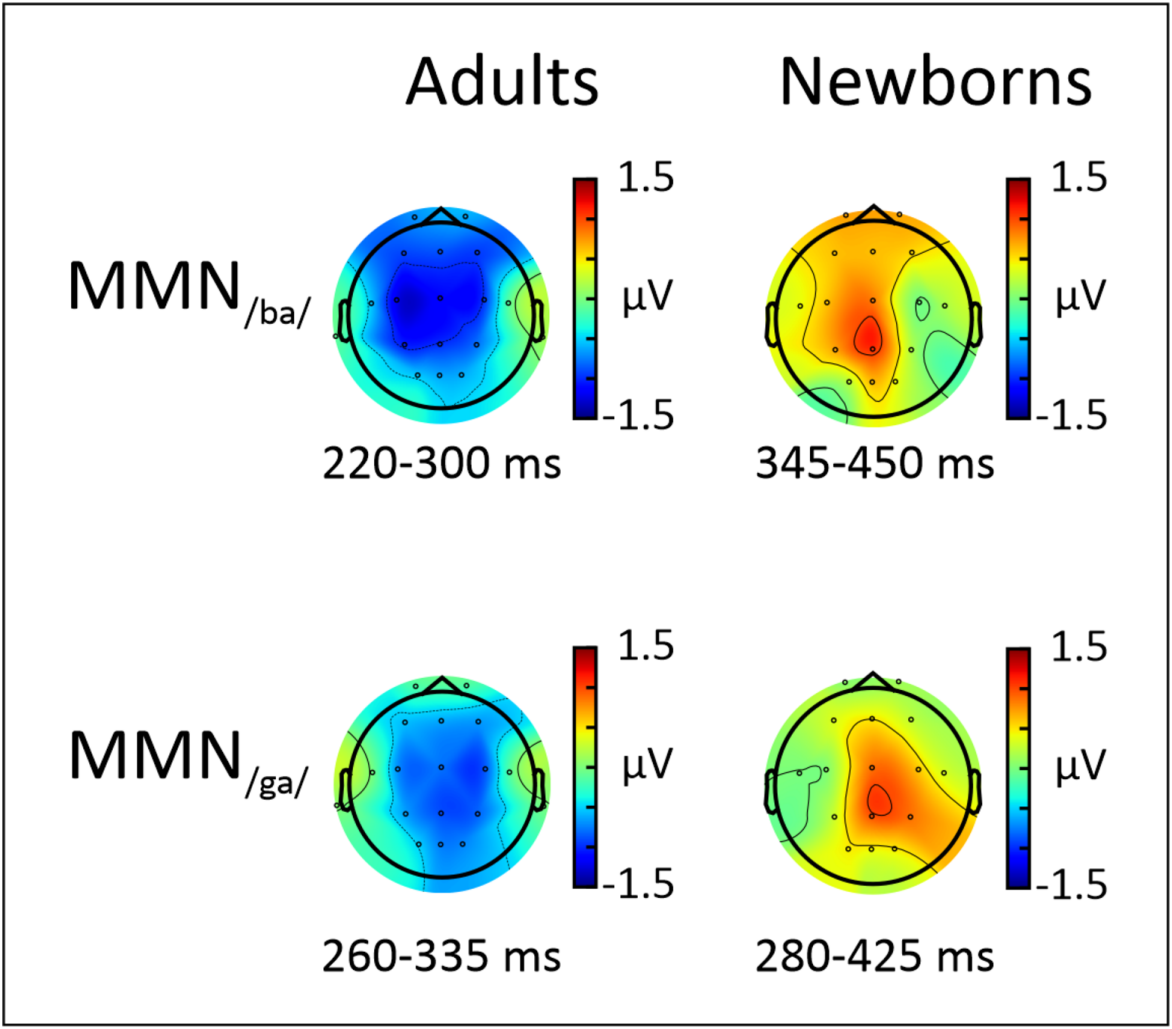
Topographical maps of the MMN mean amplitudes for the/ba/ (top) and/ga/deviant (bottom) in adults (left) and newborns (right). The time windows used for the topographies are based on the significant clusters found in the cluster‐based permutation analysis comparing standard and deviants.

Grand‐average ERPs to standard and deviants are shown at Cz and Pz electrodes separately for the two groups in Figure 5 (see also Figure S1). In adults, the two deviants elicited a larger negativity than standards between 200 and 300 ms. This observation was confirmed by the cluster‐based permutation test, showing one to two significant TWs for the/ba/deviant across all electrodes. For the/ga/deviant, significant TWs were found at Cz, C3, and Pz (see Figures 5, 6). In newborns, we found two significant clusters at Pz electrode for both types of deviants (see S1–S2 for a summary of the results obtained from the cluster‐based permutation tests performed for each group, deviant type, and electrode).

**FIGURE 7:**
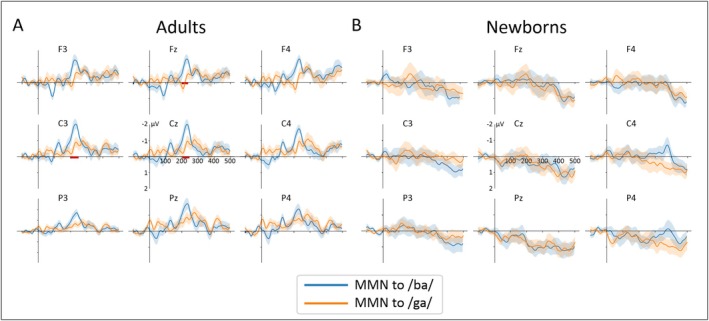
Grand‐average difference waveforms in adults (left) and newborns (right) separately for each deviant condition. MMNs to/ba/ deviant are depicted in blue whereas MMNs to/ga/deviant are depicted in orange. The shaded areas around the curves depict the standard errors. The red horizontal bars show the temporal clusters with significant differences between conditions at *p* < 0.05 corrected for multiple comparisons.

As a second step, we assessed the effect of deviant type on MMN amplitude for each group separately using cluster‐based permutation test. As can be seen on Figure 7 showing the comparison of the two types of MMN in each group, adults exhibited a significant cluster where the MMN to/ba/deviants was significantly larger than the MMN to/ga/deviants over Fz, Cz, and C4 electrodes. By contrast, no differences were found between the two conditions in newborns (see Figure 7 and Table S3).

**FIGURE 8:**
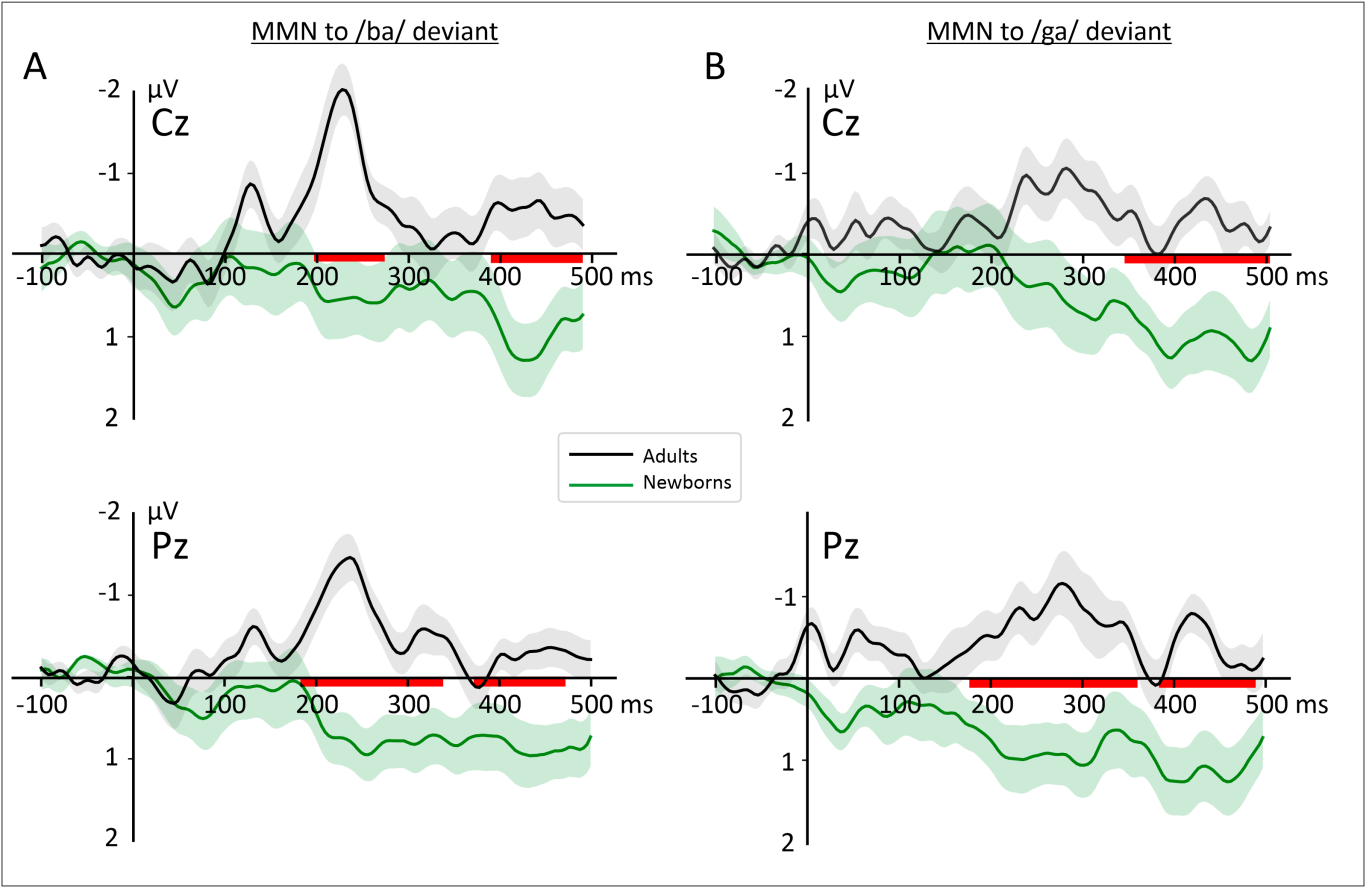
Grand‐average difference waveforms (deviant—standard) for the two groups (black: adults, green: newborns) over Cz and Pz electrodes separately for each condition (left: /ba/ deviant, right: /ga/deviant). The shaded areas around the curves depict the standard errors. The red horizontal bars show the temporal clusters with significant between‐group differences at *p* < 0.05 corrected for multiple comparisons.

Finally, we assessed differences in MMN amplitude between groups using a cluster‐based permutation test separately for each deviant (see Figure 8 and Figure S2). For the/ba/ MMN, we found two significant TWs spread across almost all electrodes. Additionally, we observed significant differences in multiple TWs for the/ga/MMN, but only across Cz, C4, Pz, and P4 electrodes (see Table S4 for a summary of the results obtained from the cluster‐based permutation tests performed for each deviant type and electrode).

## 4 Discussion

In the present study, we provide new evidence for the role of language experience on the brain machinery underlying the emergence of fine‐grained acoustic–phonetic processing. We collected cortical and subcortical brain responses to acoustically controlled speech sounds from 21 adults and 17 newborns during a single experimental session. Specifically, we used an innovative experimental procedure adapted from Bidelman (2015) that allowed us to analyze the FFRs and the MMN in a within‐participant design over a short period. To elicit the FFR, we used a synthetic/oa/suitable for evaluating neural phase‐locking to both the fundamental frequency and the vowels' first formants in newborns and adults. FFR data revealed that newborns exhibit efficient neural phase‐locking to the fundamental frequency while the encoding of the first formant remains relatively immature. The ERPs obtained during the oddball blocks revealed large frontocentral MMNs to the/da/−/ba/and −/ga/contrasts in adults. In newborns, the cortical responses were more focally distributed over parietal electrodes with positive MMRs to both deviants. These results not only replicate previous electrophysiological findings but also provide new evidence on the complex interactions between acoustic–phonetic salience, sensorimotor information, and auditory maturational mechanisms during early development.

Regarding the FFR data, our results partly replicate findings from a previous study comparing newborns and adults using the same stimulus (Arenillas‐Alcón et al. 2021). Specifically, we confirmed that newborns exhibited a slower neural transmission velocity than adults, which further highlights the role of myelination and experience‐dependent factors in shaping the precise timing of the FFR_ENV_ (Anderson et al. 2015; Kraus and Chandrasekaran 2010). However, FFR indices related to F0 encoding indicated an already functional tracking of pitch just a few days after birth. Specifically, we found that while the absolute F0 spectral amplitude was significantly higher in adults than in newborns, the relative magnitude of the response (i.e., SNR) did not differ between the groups. This stronger response in absolute spectral amplitude is likely to be explained by differences in the level of neural noise estimated with the RMS prestimulus, as also reported in the inspiring work of Arenillas‐Alcón et al. (2021). Moreover, while previous work comparing adults and newborns did not report differences in pitch error or pitch strength measures (Arenillas‐Alcón et al. 2021), we observed significant between‐group differences in the dynamic encoding of F0. Specifically, we found that newborns exhibited significantly higher pitch error than adults in the rising but not in the stable portion of the stimulus. This higher pitch error was accompanied by a significantly lower pitch strength in newborns than in adults. These observations reinforce the interpretation of a functional although still immature neural tracking of pitch at birth (Ribas‐Prats et al. 2019, 2022; Arenillas‐Alcón et al. 2021; Jeng et al. 2011, 2016). Such a difference might reflect a possible linguistic specialization of pitch encoding in the context of prosodic phonology of French which is characterized by final pitch rising patterns (Jun and Fougeron 2002).

Analyses of the FFR_TFS_ revealed further similarities between newborns and adults, with a significant effect of stimulus section in both groups, differing from the results reported in Arenillas‐Alcón et al. (2021). Interestingly, recent evidence from the same group demonstrated the impact of linguistic and musical prenatal experience on the neonatal encoding of acoustic features of speech sounds (Arenillas‐Alcón et al. 2023; Gorina‐Careta et al. 2024). Indeed, despite the low‐pass filtering induced by the womb during the prenatal period (Abrams and Gerhardt 2000; Gerhardt and Abrams 2000; Lecanuet and Schaal 1996), the fetal auditory system is fully functional, leaving room for fetal learning mechanisms (Hervais‐Adelman and Townsend 2025). Moreover, the extended experience with phonetic linguistic diversity, as in the case of multilingual families or with language‐specific phonetic environment, may also contribute to shaping the auditory brain (Kepinska et al. 2023, 2025). In French, only two types of /o/ sounds are described in the phonetic repertoire (Chollet and Malecot 1980). In contrast, Catalan is characterized by a larger set of regional variations of the /o/ sound than Spanish and French (Carbonell and Llisterri 1992). These cross‐linguistic typological differences may at least partly explain why our pattern of results showing a significant group by stimulus section interaction for the spectral amplitude of the /o/ F1 and not for the /a/ F1 is not similar to the observations reported by Arenillas‐Alcón et al. (2021). Further exciting work is needed to disentangle the role of phonetic linguistic diversity during the prenatal period on the neonatal subcortical encoding of speech sounds.

Importantly, in addition to recording the FFR to the/oa/ stimulus, our paradigm allowed us to collect ERPs to CV syllables during oddball blocks. Specifically, we used acoustically controlled CV syllables that differed in place of articulation related to the perception of dental‐bilabial (/da/ vs. /ba/) and dental‐uvular (/da/ vs. /ga/) consonantal contrasts. According to the acoustic salience hypothesis proposed by Narayan (2019), these two contrasts may not exhibit the same level of acoustic salience as/ba/ is more perceptually distant from/da/ compared to/ga/. This perceptual distance is reflected in the F2 dynamics values (see Table 1), showing an increase in F2 during the formant transition for/ba/ and not for/ga/. This difference in F2 dynamics during CV transition may thus transfer into larger and earlier MMNs to/ba/ than to/ga/ deviants in adults, while newborns do not show such a perceptual distance effect. Moreover, while the adult MMNs had a clear frontocentral topography, the newborns showed a positive MMR restricted to parietal electrodes. As previously reported, these differences in topography and polarity may reflect an immature response at this very young stage (Govaart et al. 2023). The absence of a frontocentral distribution in newborns could also reflect the limited sensorimotor articulatory experience related to the production of these contrasts, thus leaving the acoustic–phonetic differences alone in eliciting the MMRs. While the syllable/da/ involves a tip of the tongue movement, the syllable/ba/ and/ga/rely on a labial and back of the tongue movement, respectively. Interestingly, infants' production of uvular/g/ has been reported to emerge later and less frequently than/b/ and/d/ stop consonants during early development (Morgan and Wren 2018). Besides, previous behavioral and EEG studies in preverbal infants have pointed to an important role of sensorimotor articulatory information on the early perception of phonetic contrasts based on place of articulation (Bruderer et al. 2015; Choi et al. 2021, 2023). Specifically, the authors evaluated the discrimination of a non‐native dental contrast (Hindi dental‐dental retroflex contrast), while infants had a teething toy designed to block the tongue movements required to produce this contrast. Both behavioral and EEG data revealed that infants exhibited impaired discrimination of the contrast when the tongue movements were impeded.

There are some limitations to the present study. First, the neurophysiological differences observed between newborns and adults may relate to the different cortical states for adults and newborns during the experiment. Indeed, previous studies in adults have shown that cortical alpha power can modulate the amplitude of the speech‐evoked FFR (Lai et al. 2022). Similarly, there is still a debate as to whether sleep stages can modulate the polarity of the MMN in infants, with awake infants exhibiting negative MMN more frequently than asleep infants (see Govaart et al. 2023 for a recent review). In the present study, while newborns were sleeping in their crib, adult participants were awake watching a silent movie, which may induce different attentional levels and cortical states. Second, due to the limited time during which newborns could be tested, we used a rather small number of deviant trials, which is lower than in classical MMN paradigms. This small number of trials may translate into a relatively low SNR, although recent evidence has demonstrated the feasibility of extracting reasonable MMNs and FFRs to CV syllables with 50 and 200 trials, respectively (Cheng and Zhao 2024).

## 5 Conclusion

Overall, despite a relatively small sample, the present study provides new converging evidence that newborns younger than 3 days of age exhibit an immature neural encoding of the first formant of consonants and vowels while exhibiting an already functional encoding of vowel pitch. These results highlight the importance of gathering electrophysiological data evaluating the neonatal brain sensitivity to more diverse acoustic–phonetic cues to better understand the subtle linguistic differences associated with the emergence of phonological categories during infancy (Chládková and Paillereau 2020). Moreover, these results strongly suggest that linguistic and sensorimotor experience play a central role in the emergence of robust acoustic–phonetic processing of speech sounds. Finally, our data confirm the feasibility of obtaining an exhaustive snapshot of human auditory neurophysiology based on the combined analysis of the FFR and MMN in a within‐participant design.

## Author Contributions

**Giulia Danielou:** data curation (lead), formal analysis (lead), investigation (lead), writing – original draft (lead). **Estelle Hervé:** data curation (supporting), writing – review and editing (supporting). **Anne‐Sophie Dubarry:** data curation (supporting), methodology (supporting). **Béatrice Desnous:** resources (supporting). **Clément François:** conceptualization (lead), funding acquisition (lead), project administration (lead), resources (lead), supervision (lead), writing – original draft (equal), writing – review and editing (lead).

## Conflicts of Interest

The authors declare no conflicts of interest.

## Supporting information


**Figure S1:** Grand‐average ERPs to standard, deviants and to the difference waveform (Deviant–Standard) in adults (left) and newborns (right) separately for each deviant (top: /ba/deviant, bottom: /ga/deviant). ERPs to standards are shown in dark colors (dark black for adults and dark green for newborns). ERPs to deviants are shown in light colors (light gray for adults and light green for newborns) for the/ba/ (top) and/ga/deviant (bottom), respectively. The shaded areas around the curves depict the standard errors.
**Figure S2:** Grand‐average ERPs to the difference waveform (Deviant–Standard) in adults (black) and newborns (green) separately for each deviant (left: /ba/deviant, right: /ga/deviant). The shaded areas around the curves depict the standard errors. Significant clusters showing between‐group differences obtained in the cluster‐based permutation test are depicted in gray. The red horizontal bars show the temporal clusters with significant between‐group differences at *p* < 0.05 corrected for multiple comparisons.

## Data Availability

The data are not publicly available due to privacy or ethical restrictions.
