# Supplementary material for: Event‐Related Brain Potentials and Frequency‐Following Response to Syllables in Newborns and Adults
Source: Eur J Neurosci. 2026 Feb 8;63(3):e70418. doi: 10.1111/ejn.70418 (PMC12884137; doi:10.1111/ejn.70418)
Supplement: Supplementary file 1 — Figure S1: Grand‐average ERPs to standard, deviants and to the difference waveform (Deviant–Standard) in adults (left) and newborns (right) separately for each deviant (top: /ba/deviant, bottom: /ga/deviant). ERPs to standards are shown in dark colors (dark black for adults and dark green for newborns). ERPs to deviants are shown in light colors (light gray for adults and light green for newborns) for the/ba/ (top) and/ga/deviant (bottom), respectively. The shaded areas around the curves depict the standard errors. Figure S2: Grand‐average ERPs to the difference waveform (Deviant–Standard) in adults (black) and newborns (green) separately for each deviant (left: /ba/deviant, right: /ga/deviant). The shaded areas around the curves depict the standard errors. Significant clusters showing between‐group differences obtained in the cluster‐based permutation test are depicted in gray. The red horizontal bars show the temporal clusters with significant between‐group differences at p < 0.05 corrected for multiple comparisons. [file EJN-63-0-s001.docx]

**Supplementary materials**

**Danielou et al.**


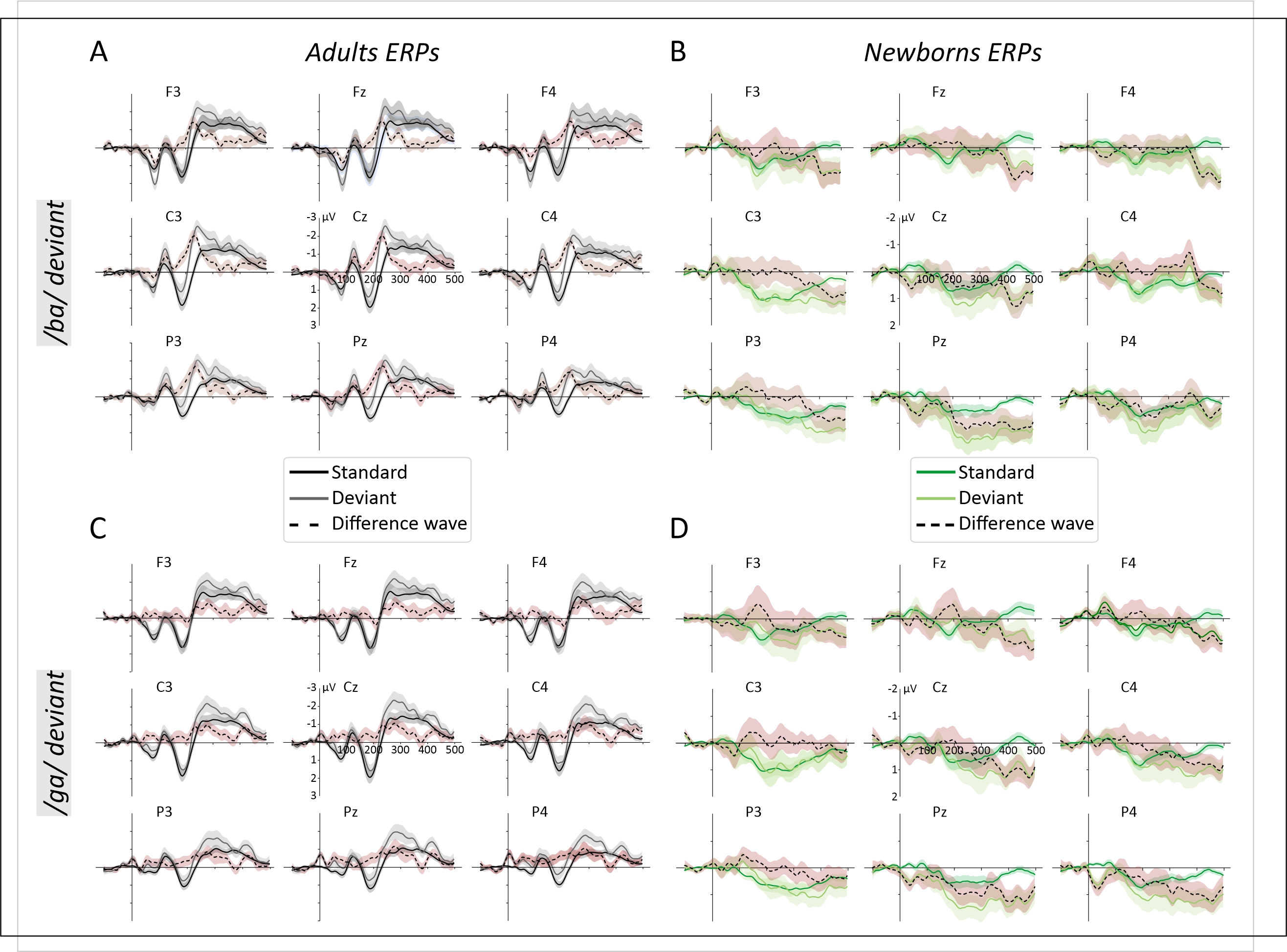


**Supplementary Figure 1:** Grand-average ERPs to standard, deviants and to the difference waveform (Deviant – Standard) in adults (left) and newborns (right) separately for each deviant (top : /ba/ deviant, bottom : /ga/ deviant). ERPs to standards are shown in dark colors (dark black for adults and dark green for newborns). ERPs to deviants are shown in light colors (light grey for adults and light green for newborns) for the /ba/ (top) and /ga/ deviant (bottom), respectively. The shaded areas around the curves depict the standard errors.


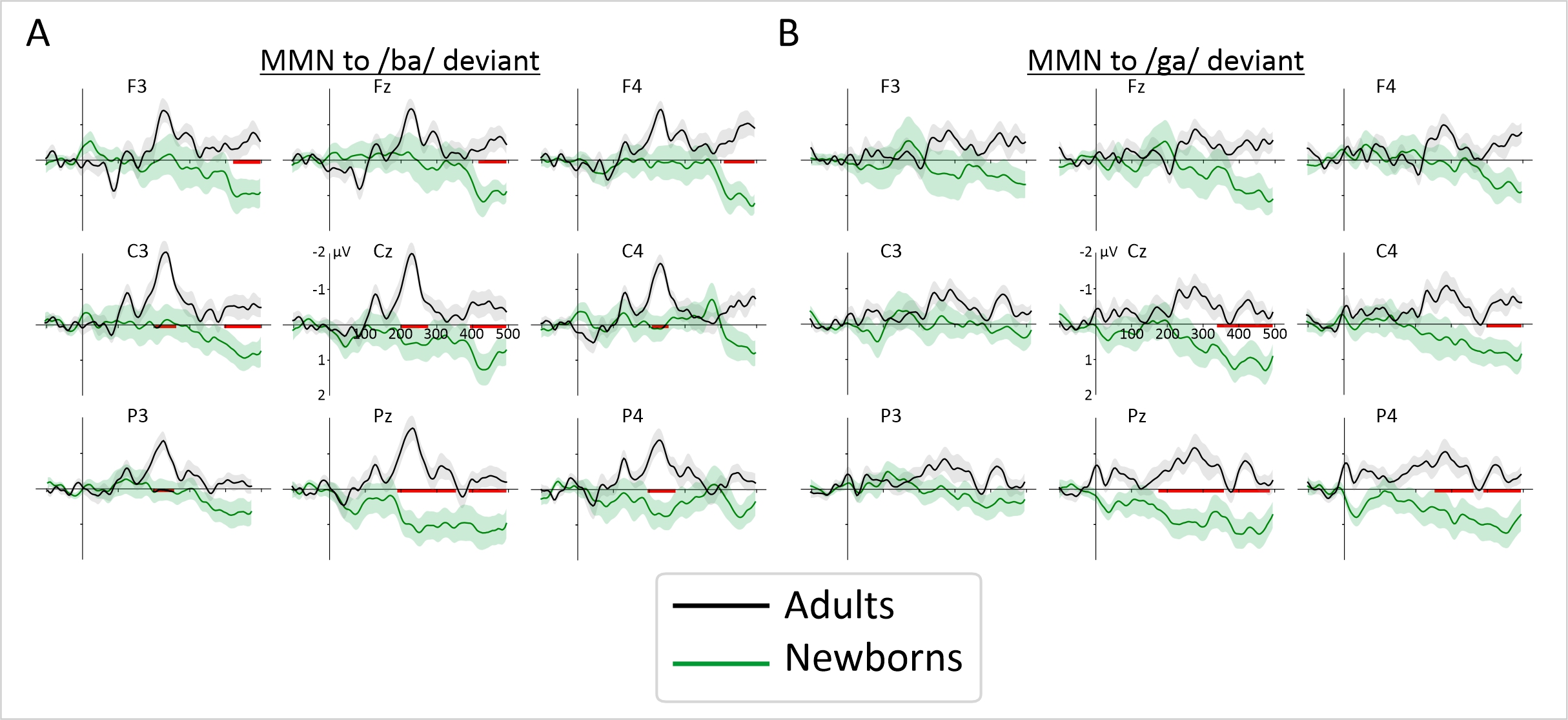


**Supplementary Figure 2:** Grand-average ERPs to the difference waveform (Deviant – Standard) in adults (black) and newborns (green) separately for each deviant (left : /ba/ deviant, right : /ga/ deviant). The shaded areas around the curves depict the standard errors. Significant clusters showing between-group differences obtained in the cluster-based permutation test are depicted in grey. The red horizontal bars show the temporal clusters with significant between-group differences at P < .05 corrected for multiple comparisons.

|  | **Standard vs. /ba/ deviant** | | **Standard vs. /ga/ deviant** | |
| --- | --- | --- | --- | --- |
| **Electrodes** | Significant clusters (ms) | *P-values* | Significant  clusters (ms) | *P-values* |
| **F3** | 211 – 301 | .003 | - | - |
| **Fz** | 211 – 258 | .009 | - | - |
| **F4** | 207 – 309  445 – 496 | .001  .021 | - | - |
| **C3** | 188 – 281 | .001 | 223 – 305 | .016 |
| **Cz** | 199 – 281 | .001 | 227 – 301 | .023 |
| **C4** | 195 – 277 | .001 | - | - |
| **P3** | 195 – 273 | .001 | - | - |
| **Pz** | 191 – 270 | .002 | 215 – 293  402 – 441 | .005  .022 |
| **P4** | 195 – 270 | .008 | - | - |

**Supplementary Table 1**: Summary of the cluster-based permutation test in adults comparing the standard /da/ to the deviant /ba/ and /ga/ separately.

|  | **Standard vs. /ba/ deviant** | | **Standard vs. /ga/ deviant** | |
| --- | --- | --- | --- | --- |
| **Electrodes** | Significant clusters (ms) | *P-values* | Significant  clusters (ms) | *P-values* |
| **F3** | - | - | - | - |
| **Fz** | - | - | - | - |
| **F4** | 422 – 496 | .037 | - | - |
| **C3** | - | - | - | - |
| **Cz** | - | - | - | - |
| **C4** | - | - | - | - |
| **P3** | - | - | - | - |
| **Pz** | 203 – 359  410 – 496 | .002  .018 | 203 – 313  371 – 488 | .009  .004 |
| **P4** | - | - | - | - |

**Supplementary Table 2**: Summary of the cluster-based permutation test in newborns comparing the standard /da/ to the deviant /ba/ and /ga/ separately.

|  | **Adults**  **(MMN_/ba/_ vs. MMN_/ga/_)** | | **Newborns**  **(MMN_/ba/_ vs. MMN_/ga/_)** | |
| --- | --- | --- | --- | --- |
| **Electrodes** | Significant clusters (ms) | *P-values* | Significant  clusters (ms) | *P-values* |
| **F3** | - | - | - | - |
| **Fz** | 199 – 238 | .04 | - | - |
| **F4** | - | - | - | - |
| **C3** | 199 – 250 | .04 | - | - |
| **Cz** | 203 – 246 | .04 | - | - |
| **C4** | - | - | - | - |
| **P3** | - | - | - | - |
| **Pz** | - | - | - | - |
| **P4** | - | - | - | - |

**Supplementary Table 3**: Summary of the cluster-based permutation test comparing the difference waveform (standard – deviant) between deviants (/ba/ vs. /ga/) in each group separately.

|  | **/ba/ deviant**  **(Adults vs. Newborns)** | | **/ga/ deviant vs. zero**  **(Adults vs. Newborns)** | |
| --- | --- | --- | --- | --- |
| **Electrodes** | Significant clusters (ms) | *P-values* | Significant  clusters (ms) | *P-values* |
| **F3** | 422 – 496 | .035 |  |  |
| **Fz** | 418 – 496 | .024 |  |  |
| **F4** | 410 – 496 | .003 |  |  |
| **C3** | 203 – 262  395 – 496 | .028  .016 |  |  |
| **Cz** | 199 – 277  395 – 496. | .010  .015 | 340 – 496 | .006 |
| **C4** | 207 – 254  441 – 496 | .020  .042 | 398 – 496 | .011 |
| **P3** | 207 – 270 | .032 |  |  |
| **Pz** | 191 – 356  391 – 496 | .001  .016 | 176 – 359  383 – 488 | .006  .011 |
| **P4** | 195 – 273 | .010 | 254 – 363  391 – 496 | .019  .016 |

**Supplementary Table 4**: Summary of the cluster-based permutation test comparing the difference waveform (standard – deviant) between groups in the /ba/ and /ga/ conditions separately.
